# Cognitive impairment in COPD: a systematic review*

**DOI:** 10.1590/S1806-37132015000004424

**Published:** 2015

**Authors:** Irene Torres-Sánchez, Elisabeth Rodríguez-Alzueta, Irene Cabrera-Martos, Isabel López-Torres, Maria Paz Moreno-Ramírez, Marie Carmen Valenza

**Affiliations:** University of Granada, Health Sciences Faculty, Department of Physical Therapy, Granada, Spain. Department of Physical Therapy, Health Sciences Faculty, University of Granada, Granada, Spain; University of Granada, Health Sciences Faculty, Department of Physical Therapy, Granada, Spain. Department of Physical Therapy, Health Sciences Faculty, University of Granada, Granada, Spain; University of Granada, Health Sciences Faculty, Department of Physical Therapy, Granada, Spain. Department of Physical Therapy, Health Sciences Faculty, University of Granada, Granada, Spain; University of Granada, Health Sciences Faculty, Department of Physical Therapy, Granada, Spain. Department of Physical Therapy, Health Sciences Faculty, University of Granada, Granada, Spain; University of Granada, Health Sciences Faculty, Department of Physical Therapy, Granada, Spain. Department of Physical Therapy, Health Sciences Faculty, University of Granada, Granada, Spain; University of Granada, Health Sciences Faculty, Department of Physical Therapy, Granada, Spain. Department of Physical Therapy, Health Sciences Faculty, University of Granada, Granada, Spain

**Keywords:** Pulmonary disease, chronic obstructive, Mild cognitive impairment, Hypoxia, brain

## Abstract

The objectives of this study were to characterize and clarify the relationships between the various cognitive domains affected in COPD patients and the disease itself, as well as to determine the prevalence of impairment in the various cognitive domains in such patients. To that end, we performed a systematic review using the following databases: PubMed, Scopus, and ScienceDirect. We included articles that provided information on cognitive impairment in COPD patients. The review of the findings of the articles showed a significant relationship between COPD and cognitive impairment. The most widely studied cognitive domains are memory and attention. Verbal memory and learning constitute the second most commonly impaired cognitive domain in patients with COPD. The prevalence of impairment in visuospatial memory and intermediate visual memory is 26.9% and 19.2%, respectively. We found that cognitive impairment is associated with the profile of COPD severity and its comorbidities. The articles reviewed demonstrated that there is considerable impairment of the cognitive domains memory and attention in patients with COPD. Future studies should address impairments in different cognitive domains according to the disease stage in patients with COPD.

## Introduction

The hallmark of COPD is chronic airflow obstruction that has a systemic impact and a progressive evolution.^(^ 1 ^)^ It is an important health problem that is estimated to become the fifth leading cause of disability and the third leading cause of death worldwide by 2020.^(^ 2 ^)^ The prevalence of COPD in the global population is close to one percent and increases with age. Among individuals 40 years of age or older in the city of São Paulo, Brazil, its prevalence ranges from 6 to 15.8%.^(^ 3 ^)^

The typical profile of patients with COPD includes multiple comorbidities,^(^ 4 ^,^ 5 ^)^ such as heart disease,^(^ 6 ^)^ osteoporosis,^(^ 7 ^)^ type 2 diabetes mellitus,^(^ 8 ^)^ lung cancer,^(^ 9 ^)^ and cognitive impairment.^(^ 10 ^)^ In recent years, the clinical relevance of cognitive impairment has risen,^(^ 11 ^)^ due to the increase in the prevalence of COPD and the growing interest in the aspects that determine functionality and treatment compliance^(^ 12 ^,^ 13 ^)^ among patients with the disease.^(^ 14 ^)^

Although COPD and cognitive impairment have been studied separately (as individual diseases), there is growing evidence of a relationship between the two.^(^ 11 ^)^ Hugg et al.^(^ 15 ^)^ analyzed cognitive impairment in patients with COPD and found that such patients had a greater risk of developing cognitive impairment than did patients without COPD. The hypoxemia seen in some patients with COPD seems to be a crucial factor for cognitive impairment, because it affects the oxygen-dependent enzymes that are important in the synthesis of neurotransmitters such as acetylcholine.^(^ 16 ^)^ Various studies have shown that cognitive impairment has a prevalence of 77% in patients with COPD and hypoxemia.^(^ 17 ^)^

The main hypotheses of this review were that there is a relationship between the various cognitive domains affected in COPD patients and the disease itself, and that the prevalence of impairment varies among the different cognitive domains. The objective of this review was two-fold: to characterize and clarify the relationship between the various cognitive domains affected in COPD patients and the disease itself; and to determine the prevalence of impairment in the various cognitive domains in such patients.

## Methods

In this review of the literature, we adopted the classification of cognitive domains devised by Lezak.^(^ 18 ^)^ According to that author, who is the current reference in neuropsychological assessment, the cognitive domains correspond to five key areas: perception; attention; memory and learning; executive function; and language. We adopted a systematic approach using the following search strings (comprising terms related to COPD and to the Lezak classification of cognitive domains): "cognitive impairment" AND "COPD"; "cognitive decline" AND "COPD"; "cognitive dysfunction" AND "COPD"; "hypoxia" AND "cognitive impairment" AND "pulmonary disease"; "cognitive impairment" AND "hypercapnia" AND "pulmonary disease"; "cognitive attention" AND "COPD"; "memory and learning" AND "COPD"; "memory learning" AND "COPD" AND "cognitive"; "perceptive function" AND "COPD"; "verbal language" AND "COPD"; and "executive functions" AND "COPD".

**Figure 1 -:**
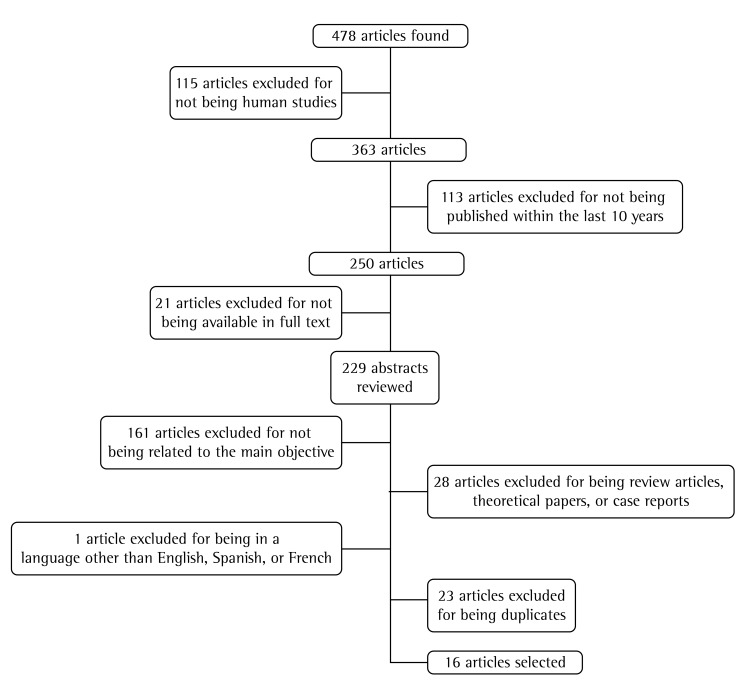
Selection of the articles analyzed in this review.

We systematically searched the following databases: PubMed, Scopus, and ScienceDirect. Searches were limited to studies in humans published in the last ten years in order to focus on the recent interest and scientific evidence in this area. The inclusion criteria were being a clinical trial, epidemiological study, observational study, cohort study, or case-control study; and providing information on the subject at hand (i.e., cognitive impairment in COPD patients). We excluded articles that dealt with subjects unrelated to this topic, those that were not available in full text, and those that were review articles or simple case reports, as well as those published in languages other than English, Spanish, or French. The article selection process is depicted as a flowchart in Figure 1.

## Results

The search yielded 478 articles. After the abstracts had been reviewed, only 16 articles were selected for inclusion in the review. The characteristics of the selected articles are shown in Table 1. Our review of those studies revealed a significant relationship between COPD and cognitive impairment. It is important to point out that there is as yet no consensus regarding the definition of cognitive impairment in patients with COPD. Different operational definitions of such impairment among the studies reviewed made it difficult to evaluate that aspect across those studies.

**Table 1 -:** Characteristics of the articles selected.

Study	Design	Objective	Sample size and COPD severity	Characteristics of COPD participants	Intervention	Results/Conclusions
Dodd et al.^(19)^	Observational study	To assess neuropsychological performance in COPD patients hospitalized after an acute exacerbation and recovery, compared with patients with stable COPD and with healthy control subjects	110 participants:		-	In patients hospitalized with an acute COPD exacerbation, impaired cognitive function is associated with worse health status and longer length of hospital stay. Cognitive function might not improve with recovery
			30 COPD inpatients hospitalized following an exacerbation	Mean age, 70 ± 11 years; 15 (50%) were female		
			50 outpatients with stable COPD	Mean age, 69 ± 8 years; 28 (56%) were female		
			30 healthy control subjects			
Chang et al.^(20)^	Cohort study	To determine the extent to which the co-occurrence of COPD and cognitive impairment leads to adverse health outcomes in older adults	3,093 patients:		None	Patients with COPD and cognitive impairment had the highest rates of respiratory-related and all-cause hospitalizations and death
			431 with COPD only	188 (43.7%) were 65-70 years of age; 210 (48.7%) were female		
			29 with COPD and cognitive impairment	6 (21.3%) were 65-70 years of age; 10 (34.5%) were female		
			114 with cognitive impairment only			
			2,519 with neither COPD nor cognitive impairment			
Dodd et al.^(21)^	Observational study	To evaluate whether there are significant differences between COPD patients and control subjects, in terms of white matter integrity and communication between gray matter resting-state networks, and to test the observed differences related to disease severity, comorbid cerebrovascular disease, and cognitive dysfunction	25 non-hypoxemic COPD patients	Mean age, 67.8 ± 8.1 years; 11 (44%) were female	None	In stable, non-hypoxemic COPD, there is reduced white matter integrity throughout the brain and widespread disturbance in the functional activation of gray matter, which might contribute to cognitive dysfunction. White matter microstructural integrity is independent of smoking and comorbid cerebrovascular disease, but gray matter functional activation is not. The mechanisms remain unclear but could include cerebral small vessel disease caused by COPD
			25 control subjects			
Villeneuve et al.^(10)^	Observational study	To determine the frequency and subtypes of MCI in COPD patients and to assess the validity of two cognitive screening tests (the MMSE and MoCA) in detecting MCI in COPD patients	45 patients with moderate-to-severe COPD	Mean age, 68.84 ± 8.43 years; 29 (64%) were female	None	In this preliminary study, a substantial proportion of COPD patients were found to have MCI. The MoCA was better than was the MMSE at detecting MCI in COPD patients.
			50 healthy control subjects			
Martin et al.^(22)^	Clinical trial	To determine the effect of hypoxia on cognitive performance in COPD patients with PaO_2_ <6.6 kPa	10 patients with moderate-to-severe COPD	Mean age, 64 years; 3 (30%) were female	For a short period of time, patients breathed 21% O_2_ when PaO_2_ was < 6.6 kPa	Short-term exposure to hypoxia had no adverse effect on cognitive function
Pereira et al.^(23)^	Clinical trial	To evaluate the effect of a multidisciplinary pulmonary rehabilitation program on cognitive function in COPD patients, adjusting for potential confounders	34 patients with moderate-to-severe COPD	Mean age, 65.2 ± 7 years; 17 (50%) were female	3-month program of pulmonary rehabilitation	Even after adjusting for the sociodemographic factors that might affect cognitive function, the authors found that pulmonary rehabilitation improved cognitive performance in COPD patients. There were gender- and age-related differences in cognitive scores that persisted after rehabilitation
			18 healthy control subjects			
Klein et al.^(24)^	Cohort study	To explore the influence of COPD on attentional functions, learning, and logical thinking	60 COPD patients	Mean age, 63.2 ± 9.8 years; 24 (40%) were female	None	In COPD patients, there was global impairment in cognitive functions that was negatively influenced by advancing age and increased in proportion to the degree of disease severity
			60 control subjects			
Thakur et al.^(25)^	Cohort study	To elucidate the association between COPD and the risk of cognitive impairment, in comparison with control subjects without COPD	1,202 COPD patients	Mean age, 58.2 ± 6.2 years; 691 (57.4%) were female	None	COPD is a major risk factor for cognitive impairment. In COPD patients, hypoxemia is a major contributor to cognitive impairment and regular use of home oxygen is a protective factor. Health care providers should consider screening COPD patients for cognitive impairment
			302 control subjects			
Antonelli-Incalzi et al.^(14)^	Observational study	To assess whether certain neuropsychological patterns are associated with various limitations to physical independence in COPD patients	149 COPD patients	112 (75.2%) were over 65 years of age; 14 (9.4%) were female	None	Classic indicators of the severity of COPD showed no correlation with personal autonomy
Borson et al.^(26)^	Observational study	To model the relationship between respiratory failure and domains related to brain function, including low mood, subtly impaired cognition, systemic inflammation, and structural/neurochemical brain abnormalities	9 healthy control subjects		None	COPD is associated with slight decreases in mood and cognition. Severe COPD is associated with chronic systemic inflammation and subtle cognitive deficits (on digit symbol coding tasks). Levels of oxygen desaturation appear to mediate specific changes in brain neurochemistry and structure that suggest sustained brain damage
			18 COPD patients, half of whom were oxygen-dependent	Mean age, 68.5 ± 8.0 years; 11 (64%) were female		
Orth et al.^(27)^	Observational study	To analyze driving performance in COPD patients and healthy control subjects	17 COPD patients	Mean age, 55.2 ± 9.3 years	None	Compared with healthy control subjects, COPD patients are more likely to cause a traffic accident. Impaired driving performance in COPD patients cannot be predicted on the basis of the severity of the disease
			10 healthy control subjects			
Pinto de Lima et al.^(28)^	Observational study	To test the hypothesis that clinically stable COPD patients without overt cognitive symptoms can nonetheless have subtle cognitive impairment	30 COPD patients	Mean age, 65 ± 8 years; 10 (33%) were female	None	There might be subclinical encephalopathy in COPD, characterized by subtle impairment of global cognitive ability
			34 control subjects	24 (71%) were female		
Salik et al.^(29)^	Observational study	To determine the relationship between cognitive function and quality of life in COPD patients with mild hypoxemia and moderate airway obstruction	32 patients with moderate stable COPD	Mean age, 66.7 ± 2.5 years; 14 (44%) were female	None	Cognitive function in COPD patients with hypoxemia might not be impaired despite their poor quality of life status
			26 healthy subjects			
Antonelli-Incalzi et al.^(30)^	Observational study	To evaluate the prognostic role of cognitive impairment in patients with severe COPD	149 COPD patients who had undergone a period of in-hospital rehabilitation following an acute exacerbation	Mean age, 68.7 ± 8.5 years; 22 (16.4%) were female	None	Impaired drawing ability is a risk factor for mortality and its testing might improve the assessment of hypoxemic COPD patients
Corsonello et al.^(31)^	Observational study	To determine whether cancer is more disabling than are other chronic diseases that are highly prevalent in the elderly	6 groups of patients:		None	Cognitive impairment was more prevalent in patients with congestive heart failure or COPD than in those with cancer
			Congestive heart failure (n = 832)			
			Diabetes mellitus (n = 939)			
			COPD (n = 399)	178 (44.6%) were 65-79 years of age; 147 (36.8%) were female		
			Non-metastatic solid tumors (n = 813)			
			Metastatic solid tumors (n = 259)			
			Leukemia/lymphoma (n = 326)			
Antonelli-Incalzi et al.^(32)^	Observational study	To determine whether the neuropsychological performance of untreated patients with OSA conforms to a distinctive pattern	49 newly diagnosed, untreated OSA patients		None	A minority of newly diagnosed OSA patients had distinct neuropsychological impairment. The greater body mass index of cognitively impaired OSA patients indicates that the metabolic syndrome might also be causally related to the cognitive dysfunction
			27 patients with multi-infarct dementia			
			31 patients with mild-to-moderate dementia of the Alzheimer type			
			63 patients with severe COPD			

MMSE: Mini-Mental State Examination; MoCA: Montreal Cognitive Assessment; MCI: mild cognitive impairment; kPa: kilopascal; CHF: congestive heart failure; and OSA: obstructive sleep apnea.

Given the multiple classifications of cognitive domains and the complexity of the assessment tools available, we chose the classification proposed by Lezak,^(^ 18 ^)^ which is one of the most complete and comprehensive such classifications devised to date. Table 2 shows the various tests used and the cognitive domains assessed in the selected articles.

**Table 2 -:** Tests or batteries of tests used in the assessment of the cognitive domains under study in the articles selected.

Neuropsychological assessment instrument or function assessed	Cognitive domain						
	Perception	Attention	Memory and learning	Abstract thinking and executive function	Language	Intelligence	General (global screening)
Wechsler Test of Adult Reading^(19,22)^						X	
Mini-Mental State Examination^(10,19-21,25,28-30)^							X
Rey Complex Figure Test-Copy and Rey Complex Figure Test-Recall^(19,21,22)^			X				
Wechsler Memory Scale-III UK Word Lists^(19)^			X				
Delis-Kaplan Verbal Fluency test^(19)^					X		
Delis-Kaplan Trail Making Test^(10,19,22)^				X			
Wechsler Adult Intelligence Scale-III UK Letter-Number Sequencing^(19,21)^				X			
Wechsler Memory Scale-III UK Spatial Span^(19,23)^				X			
Wechsler Adult Intelligence Scale-III Digit Symbol^(19,26)^				X			
Wechsler Adult Intelligence Scale-III Symbol Search^(19,21)^				X			
Montreal Cognitive Assessment^(10)^							X
Digit Span Test (Wechsler Adult Intelligence Scale-III)^(10,23)^		X		X			
Digit Symbol coding test (Wechsler Adult Intelligence Scale-III)^(10)^		X		X			
Semantic Verbal Fluency^(10)^		X		X			
Letter verbal fluency (P, F and L)^(10)^		X		X			
Rey Auditory Verbal Learning Test^(10,23)^			X				
Block Design^(10)^	X						
Bells Test^(10)^	X						
Word Lists Learning, Delayed Recall, and Delayed Recognition (Wechsler Memory Scale-III)^(21)^			X				
Verbal Fluency-FAS task (Delis-Kaplan Executive Function System)^(21,23)^				X			
Stroop Color-Word Test^(10,23)^				X			
Attention Network Test^(24)^		X		X			
Standard Progressive Matrices^(24)^							
Verbal and Nonverbal Learning Test (part of the Vienna Test System)^(24)^			X				
Raven’s Colored Progressive Matrices^(14)^	X						
Phonemic verbal fluency test^(14)^					X		
Corsi Block-Tapping task (visuospatial span)^(14)^	X		X				
Verbal word span^(14)^		X	X				
Rey Auditory 15-Word Learning test^(14)^			X				
Albert’s test (visual exploration)^(14)^	X						
Copying geometrical drawings with or without landmarks^(14)^	X						
Immediate Visual Memory Test^(14)^			X				
Sentence construction^(14)^					X		
The Computer-Aided Risk Simulator (driving simulator test)^(27)^	X	X					
Dementia Rating Scale-2^(26)^							X
Wide Range Achievement Test-3^(26)^						X	
Logical memory subtest of the Wechsler Memory Scale-III^(26)^			X				
Mental Deterioration Battery^(30)^							X
10-item Hodkinson Abbreviated Mental Test^(31)^							X
							

Of the 16 studies selected, 14 were descriptive studies and two were experimental studies. Of the 14 descriptive studies, 11 were observational studies and three were cross-sectional studies. 

## Discussion

Various controlled studies have investigated the prevalence of cognitive impairment in COPD,^(^ 21 ^,^ 25 ^,^ 28 ^)^ showing that prevalence to be higher in COPD patients than in healthy control subjects.^(^ 10 ^,^ 31 ^)^ According to such studies, mild cognitive impairment is present in 36% of COPD patients and in 12% of subjects without COPD. In a study conducted by Antonelli-Incalzi et al.,^(^ 30 ^)^ the prevalence of cognitive impairment and severe cognitive impairment in COPD patients was found to be 32.8% and 10.4%, respectively. 

The prevalence of cognitive impairment in patients with COPD was found to be associated with the severity of the disease,^(^ 20 ^,^ 25 ^)^ being 3.9% among patients with mild COPD, 5.7% among patients with moderate COPD, and 7.7% among patients with severe COPD. In fact, a relationship has been found between the Mini-Mental State Examination score and the severity of COPD (r = −0.49, p < 0.001).^(^ 28 ^)^ However, the study conducted by Salik et al.^(^ 29 ^)^ showed that cognitive function in COPD patients with mild hypoxemia was similar to that observed for healthy subjects. According to those authors, cognitive function is affected by hypoxemia only when the latter is severe. In addition, Grant et al.^(^ 17 ^)^ reported a 77% prevalence of cognitive impairment in patients with hypoxemic COPD. The reasons for this variation across studies include differences in the degree of COPD severity and in the age of the patients included in the studies, as well as the use of different diagnostic criteria for cognitive dysfunction and different cognitive tests. 

The studies included in our review had large sample sizes and included a great variety of patients, which reduces any bias in prevalence rates. It is known that COPD is associated with an increased risk of impaired cognitive function,^(^ 26 ^)^ even when the data are adjusted for age, gender, smoking history, and level of education.^(^ 19 ^,^ 25 ^)^ Villeneuve et al.^(^ 10 ^)^ reported that level of education was the only variable for which there were significant differences among COPD patients with mild cognitive impairment, COPD patients without cognitive impairment, and healthy control subjects.^(^ 10 ^)^ The authors ruled out strokes and other cardiovascular diseases (all of which are common among COPD patients) as risk factors. Low peripheral oxygen saturation (≤ 88%) has been strongly associated with a risk of cognitive impairment in patients with COPD, and the use of home oxygen therapy has been associated with a reduction in that risk.^(^ 25 ^)^ Numerous studies have explored the relationship between COPD and cognitive impairment. While some studies have addressed this issue globally using screening tests, others have focused on the assessment of specific cognitive domains.^(^ 10 ^,^ 24 ^,^ 27 ^)^

Perception is a series of processes and activities through which we extract information about our environment, the actions we perform within it, and our own state. Perceiving the environment requires a proper combination of attention and perception. Therefore, even though attention and perception are considered separate areas in the Lezak classification, they are often assessed together. Hypothesizing that automobile accidents would be more common among drivers with COPD than among those without, Orth et al.^(^ 27 ^)^ compared COPD patients with healthy control subjects, in terms of complex attentional and perceptual functions. The authors found that, in simulated driving situations, COPD patients showed lower concentration values and had a significantly higher number of accidents than did the healthy control subjects.^(^ 27 ^)^ According to various studies,^(^ 10 ^,^ 24 ^)^ attentional and executive functions are commonly impaired in the main subtype of mild cognitive impairment found in patients with COPD.

Learning implies acquiring information and therefore changes the state of memory. Verbal memory is one of the cognitive domains that are most frequently impaired in patients with COPD.^(^ 31 ^)^ According to Villeneuve et al.,^(^ 10 ^)^ verbal memory and learning is the second most commonly impaired cognitive domain in patients with COPD. In such patients, the prevalence of impairment in visuospatial memory and intermediate visual memory is 26.9% and 19.2%, respectively.^(^ 14 ^)^ In patients with COPD and sleep apnea, verbal memory and visual memory are the most commonly affected cognitive domains,^(^ 30 ^)^ although processing speed, working memory, and executive function are also affected (p = 0.01, p = 0.02, and p ≤ 0.001, respectively).^(^ 21 ^)^

The term "executive functions", coined by Lezak, refers to skills involved in formulating goals, planning their achievement, and effectively performing behaviors.^(^ 18 ^)^ The assessment of executive functions in patients with COPD has shown that such patients tend to have slower processing speeds.^(^ 19 ^)^ Twenty percent of patients with exacerbated COPD exhibit a loss in processing speed that is significant enough to be considered pathological. Slower processing speed has been related to the duration of hospital stay, quality of life measured with the Saint George's Respiratory Questionnaire, and the number of COPD exacerbations.^(^ 19 ^)^

The ability to understand and communicate is determined by language. This mental process enables structured thinking, allowing an individual to make connections between ideas and mental representations. There have been studies evaluating cognitive function in a number of diseases,^(^ 30 ^)^ including sleep apnea and COPD. Patients with COPD and sleep apnea have been found to perform more poorly on tests of verbal fluency and deductive thinking than do COPD patients without sleep apnea. There are data indicating that only 3% of COPD patients have a completely normal cognitive profile.^(^ 19 ^)^

We made cognitive impairment the focus of the present review because it is a common comorbidity in patients with COPD. The strength of our review is that it explored the relationship between COPD and cognitive impairment in the various cognitive domains over the last ten years, during which time a number of relevant clinical studies on this subject have been conducted. In addition, the studies included had large sample sizes. There have been a number of reviews of cognitive impairment in elderly people and COPD patients.^(^ 33 ^-^ 36 ^)^ The review conducted by Schillerstrom et al.^(^ 33 ^)^ addressed the impact of medical illness on executive function. In another review, Dodd et al.^(^ 11 ^)^ explored the mechanisms that cause injury and dysfunction in the brain, discussing the methods used in order to evaluate cognition, gathering evidence on the nature and level of cognitive impairment in COPD. Another recent review, conducted by Schou et al.,^(^ 37 ^)^ investigated the occurrence and severity of cognitive dysfunction in COPD patients, exploring the relationship between the severity of COPD and the level of cognitive function. In our review, we included nine new studies about COPD and cognitive impairment, conducted between 2009 and 2013, which were excluded from the review conducted by Schou et al.,^(^ 37 ^)^ because they were not published within the date range set for the search of the literature in the latter.

One of the limitations of the present review is the great variety of outcome measures evaluated. However, our review of the literature clearly showed the existence of a relationship between COPD and cognitive impairment. That relationship appears to be determined by the severity of COPD and by its comorbidities. 

The most widely studied cognitive domains are memory and attention, both of which have been explored with specific assessment tools and found to be considerably impaired in patients with COPD. Evidence suggests that a structured assessment of cognitive function should be a routine component of the evaluation of COPD patients. Future studies should explore impairment in the various cognitive domains in COPD patients at different stages of the disease.
